# Longitudinal diffusion tensor magnetic resonance imaging analysis at the cohort level reveals disturbed cortical and callosal microstructure with spared corticospinal tract in the *TDP-43*^*G298S*^ ALS mouse model

**DOI:** 10.1186/s40035-019-0163-y

**Published:** 2019-08-30

**Authors:** Hans-Peter Müller, David Brenner, Francesco Roselli, Diana Wiesner, Alireza Abaei, Martin Gorges, Karin M. Danzer, Albert C. Ludolph, William Tsao, Philip C. Wong, Volker Rasche, Jochen H. Weishaupt, Jan Kassubek

**Affiliations:** 1 Department of Neurology, University of Ulm, Oberer Eselsberg 45, RKU, D-89081 Ulm, Germany; 2 German Center for Neurodegenerative Diseases (DZNE), Ulm, Germany; 3 Core Facility Small Animal MRI, University of Ulm, Ulm, Germany; 4 Department of Pathology, The Johns Hopkins University School of Medicine, Baltimore, USA

**Keywords:** Diffusion tensor imaging, Amyotrophic lateral sclerosis, Mutant *TDP-43*, Fiber tracking, Mouse brain

## Abstract

**Background:**

In vivo diffusion tensor imaging (DTI) of the mouse brain was used to identify TDP-43 associated alterations in a mouse model for amyotrophic lateral sclerosis (ALS).

**Methods:**

Ten mice with *TDP-43*^*G298S*^ overexpression under control of the Thy1.2 promoter and 10 wild type (*wt*) underwent longitudinal DTI scans at 11.7 T, including one baseline and one follow-up scan with an interval of about 5 months. Whole brain-based spatial statistics (WBSS) of DTI-based parameter maps was used to identify longitudinal alterations of *TDP-43*^*G298S*^ mice compared to *wt* at the cohort level. Results were supplemented by tractwise fractional anisotropy statistics (TFAS) and histological evaluation of motor cortex for signs of neuronal loss.

**Results:**

Alterations at the cohort level in *TDP-43*^*G298S*^ mice were observed cross-sectionally and longitudinally in motor areas M1/M2 and in transcallosal fibers but not in the corticospinal tract. Neuronal loss in layer V of motor cortex was detected in *TDP-43*^*G298S*^ at the later (but not at the earlier) timepoint compared to *wt*.

**Conclusion:**

DTI mapping of *TDP-43*^*G298S*^ mice demonstrated progression in motor areas M1/M2. WBSS and TFAS are useful techniques to localize *TDP-43*^*G298S*^ associated alterations over time in this ALS mouse model, as a biological marker.

## Background

Diffusion tensor imaging (DTI) has become an important tool to study the anatomy of the mouse brain in vivo [1, 2, 13, 30], and its non-invasive nature enables longitudinal studies of transgenic murine disease models [12, 34]. Ultra-high fields (11.7 T) and dedicated resonators (cryogenic cooled resonator-CCR) allow the recording of high-resolution DTI datasets with in-plane resolutions down to 156 μm × 250 μm with an axial slice thickness of 250 μm [27] while the development of fast DTI protocols has reduced acquisition time to 35 min, enabling the logistics for the monitoring of larger cohorts [28]. The combination of these approaches enables the study of the large-scale structural integrity of white matter and axonal tracts as well as of the microstructure of brain structures over time [28]. Moreover, DTI allows the exploration of (micro) anatomical integrity and changes in a brain-wide, unbiased and operator-independent manner.

The large-scale structure of myelinated, long-range axonal projections has recently emerged as imaging biomarker of disease progression in Amyotrophic Lateral Sclerosis (ALS): DTI investigation of the integrity of cortico-efferent axonal tracts has revealed the progressive involvement of cortico-spinal, cortico-rubral and cortico-striatal subpopulations of projection neurons in human patients [17], in agreement with the pattern of ALS-related pathobiochemistry (i.e., tar DNA binding protein 43 (TDP-43) inclusions) detected in neuropathological studies [4]. Moreover, DTI has been instrumental in comparing the patterns of anatomical involvement in ALS clinical variants [29].

Nevertheless, much less is known about the large-scale involvement of axonal projections in ALS murine models and in particular about the anatomical patterns and the overall degree of structural involvement in the brain. A comparative study of retrograde-AAV projection tracing in the *SOD1*^*G93A*^ mouse model has revealed substantial similarities between the connectivity remodeling in human patients and murine models [7]. Likewise, the loss of axons in the spinal cord has been detected in vivo by DTI approaches [22]. Very little is known about non-SOD1 models, which may recapitulate different aspects of the ALS/FTD disease spectrum. In fact, mutant *TDP-43* transgenic mice develop axonal loss and progressive cognitive phenotypes [3, 21], together with biochemical signs of *TDP-43* aggregation [43]. However, the extent of brain involvement in *TDP-43*^*G298S*^ transgenic mice has not been fully investigated because histological techniques cannot be easily implemented on every brain structure and may suffer from significant biases in quantification. Thus, the extent of involvement of the motor cortex as well as any pathological change in extra-motor areas and their progression over time remain unclear. We focused on the *TDP-43*^*G298S*^ transgenic mouse model because, in contrast to the mutant *SOD1* model, it may more faithfully mimic the pathogenic cascades and the neuropathological hallmarks observed in human patients affected by sporadic ALS, frontotemporal dementia (FTD), and related conditions [3, 4, 9]. Here, whole brain-based spatial statistics (WBSS) of DTI data [26] was applied to the *TDP-43*^*G298S*^ transgenic mouse model of ALS in order to establish the large-scale pattern of abnormalities in the cerebral microstructure in a longitudinal experimental design.

## Materials and methods

### Animals

Mice expressing full-length mutant (G298S) human *TARDBP* (hereafter referred to as *TDP-43*^*G298S*^ mice) were provided by Phil Wong (line available at the Jackson Laboratory, Stock No: 017589, Thy1.2-TDP43*G298S line S97). Mice were maintained at 22 °C with a 12/12 h light/dark cycle and had food and water ad libitum. All animal experiments were performed in accordance with institutional guidelines of Ulm University and were approved by the according regulatory authority (Regierungspräsidium Tübingen, Germany; animal permission no. 1242). Twice a week, male mice were subjected to weighing and disease scoring. Ten adult wild type (*wt*) mice and ten heterozygous *TDP-43*^*G298S*^ mice (mean age 9.3 months at baseline scan) underwent the whole-brain DTI-MRI protocol. Heterozygous *TDP-43*^*G298S*^ mice develop a robust motor neuron disease phenotype with tremor and amyotrophic paralysis of the limbs progressing to flaccid paralysis at around 2 years of age (data not shown; [39, 43]).

Data acquisition was performed under isoflurane anesthesia (5% for induction and 1.5% for maintenance). The animals were placed in a stereotactic head support (Bruker Biospin, Ettlingen, Germany) to immobilize the head. Body temperature was controlled by an integrated water-based heating device. The body temperature of the mouse was monitored by a rectal temperature probe and respiration was monitored by a respiratory pillow positioned under the abdomen of the mouse. The breathing frequency was maintained at 75–80 cycles per minute. The mice rapidly recovered (< 5 min) after the termination of anesthesia at the end of the MRI procedure.

### Data acquisition

Imaging was performed with an 11.7 T small bore animal scanner (Biospec 117/16, Bruker, Ettlingen, Germany). A two-element transmit/receive ^1^H mouse cryogenic surface coil (Cryo-Probe, Bruker BioSpin) was used for data acquisition. Imaging parameters of the optimized rapid diffusion prepared spin echo EPI imaging protocol were as: TE/TR 36.0 ms / 5000 ms, matrix 180 × 40, in-plane resolution 102 μm × 102 μm, 70 slices with a slice thickness of 250 μm. Thirty diffusion directions with b = 1000 s/mm^2^ and 5 unweighted b = 0 volumes (standard gradient scheme as provided by the Bruker software), 1 signal average, were acquired, resulting in a total acquisition time of 22 min.

The sequence was respiratory gated on a slice by slice basis. The mice underwent two scans, one baseline and one follow-up scan after a time interval of approximately 5 months. If possible, an additional scan after a further follow-up interval of 5 months was acquired. These additional scans were used to replace the follow-up scans in order to select the optimum data quality for baseline and follow-up scan. One *wt* mouse and one *TDP-43*^*G298S*^ mouse had only one scan useful for analysis, the remaining scans of these mice did not pass quality control due to motion artefacts. Thus, in summary 9 *TDP-43*^*G298S*^ mice and 9 *wt* mice, each with a baseline and one follow-up scan were used for analysis.

### Data processing

**Fig. 1:**
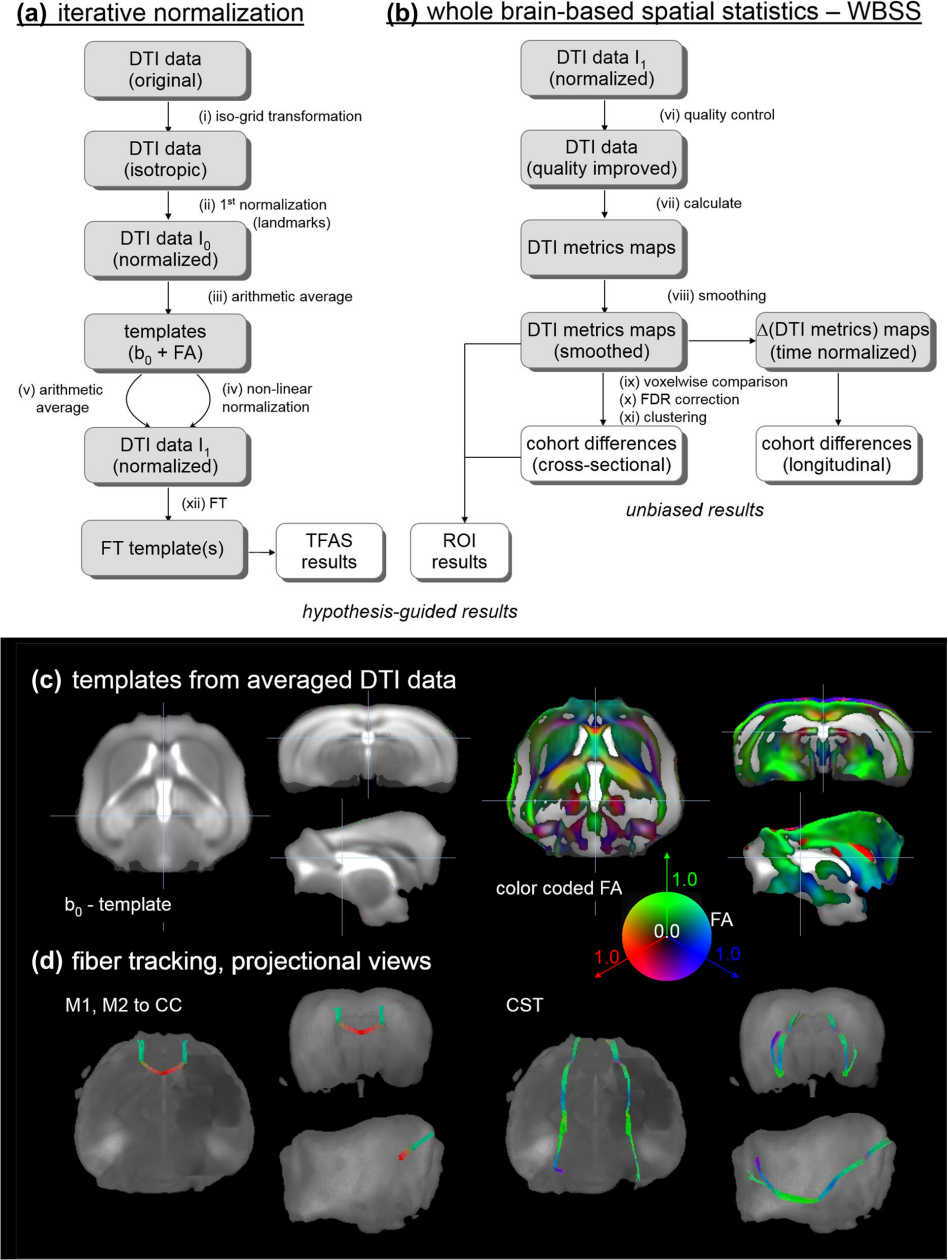
Schematic flow-chart of the iterative stereotaxic normalization during preprocessing of DTI data. **a** After transformation into a 50 μm isogrid (i), scanner- and sequence specific b_0_- and FA-templates were created in a first step by arithmetically averaging data sets of all mice after linear transformation according to manually set landmarks (ii, iii). After non-linear normalization (iv) and further arithmetic averaging (v), the template for optimized fiber tracking (FT) was use for normalization of DTI data. These FT results (xii) were used for calculating tractwise fractional anisotropy statistics (TFAS). **b** Whole brain-based spatial statistics was performed on FA maps (vii) after quality check (vi). FA maps of each data set were were smoothed with a Gaussian filter of 200 μm full-width-at-half-maximum (viii). Statistical comparisons of FA were performed voxelwise (ix). Statistical results were corrected for multiple comparisons by use of the false-discovery-rate (FDR) algorithm at *p* <  0.05 (x); further reduction of the alpha error was performed by a spatial correction algorithm (xi) to finally obtain cross-sectional and longitudinal differences at the cohort level. Illustration of quality of the DTI-based templates and tract and ROI identifications. **c** b0 and color coded FA templates. **d** FT connecting left and right motor areas M1,M2 via the corpus callosum (CC) and in the corticospinal tract (CST)

Data processing was performed with the *Tensor Imaging and Fiber Tracking* (TIFT) software package [23] which has been successfully applied to animal DTI group studies (e.g. [26, 27]). A schematic overview of the analysis cascade is provided in Fig. 1.

### Stereotaxic normalization

Recorded data were transformed into a 50 μm isogrid (nearest neighbor interpolation) in order to minimize partial volume effects. The slice thickness to in-plane resolution ratio of 1.6 as well as the recorded brain grid were in the same order as in human DTI studies since the transformation to an iso-grid of 50 μm corresponds to an iso-grid of 1 mm in human studies. Spatial normalization to a stereotaxic standard space (Fig. 1a) was performed using a study-specific b0-template and an FA-template [26] (Fig. 1c). The whole normalization process was iterative: scanner- and sequence specific b0- and fractional anisotropy (FA)-templates were created in a first step by arithmetically averaging data sets of all mice after linear transformation according to manually set landmarks identified using a stereotaxic mouse atlas [32]. After this first iteration, data were non-linearily normalized during a second iteration step in order to further optimize the normalization matrices. This process was iteratively repeated until the correlation between the individual FA-maps and the FA-template was > 0.7 which was achieved after two iterations.

Motion artefacts were eliminated in each data set separately by a dedicated quality check procedure [25] (Fig. 1b). This quality check procedure was also used to select two high-quality scans of each mouse that were compared longitudinally.

DTI-based maps (FA, axial diffusivity – AD, radial diffusivity – RD, mean diffusivity - MD) were calculated from these MNI normalized data sets. DTI metrics maps (FA, AD, RD, MD) of each data set were calculated and were smoothed with a Gaussian filter of 200 μm full-width-at-half-maximum (Fig. 1b). The filter size which is about 2–3 times the recording voxel size provides a good balance between sensitivity and specificity.

### Whole brain-based spatial statistics

Whole-brain-based spatial statistics (WBSS – [26]) was performed for cross-sectional comparison of *TDP-43*^*G298S*^ mice vs *wt* mice at baseline and at follow-up, using an FA threshold of 0.2 [20]. Statistical comparisons of DTI metrics maps for *TDP-43*^*G298S*^ vs *wt* were performed voxelwise by means of the Student’s t-test. Statistical results were corrected for multiple comparisons with the false-discovery-rate (FDR) algorithm at *p* <  0.05 [10]. Further reduction of the alpha error was performed by a spatial correction algorithm in the size range of the smoothing kernel leading to a cluster size threshold of 256 voxels.

WBSS of longitudinal DTI metrics’ map differences (ΔDM) was performed by calculating voxelwise differences between FA maps of baseline and follow-up scans for *TDP-43*^*G298S*^ mice and *wt* mice; differences were then linearly normalized to an identical time-interval prior to statistical comparison:

1 $$ \Delta \mathrm{DM}=\left(\mathrm{DM}\left({\mathrm{t}}_1\right)-\mathrm{DM}\left({\mathrm{t}}_2\right)\right)/\left({\mathrm{t}}_1-{\mathrm{t}}_2\right)\ast 1d $$

t_1_ and t_2_ are the date of baseline and follow-up scans, DM is the respective DTI metric (AD, RD, MD). Statistical comparisons of ΔDM were performed voxelwise by means of the Student’s t-test and results were corrected for multiple comparisons and also by clustering with a cluster size threshold of 256 voxels (Fig. 1b).

### Region-of-interest analyses

In order to test for hemispherical symmetry, a hypothesis-guided approach was performed: spherical regions-of-interest (ROIs) were placed in defined anatomical regions, in this study in the motor areas, in the retrosplenial cortex, in the agranular insula, and as a reference in the visual cortex. Mean DM values within the respective ROI were calculated from each single DM map.

### Tractwise fractional anisotropy statistics

An averaged data set was created from all contributing data sets, and fiber tracking (FT) was performed by deterministic streamline tracking technique [24] at an FA threshold of 0.2. In order to obtain a quantitative access to the tractography results, tractwise fractional anisotropy statistics (TFAS) [24] was applied for a hypothesis-guided analysis of FT bundles (Fig. 1d).

### Histology

For the study of histological counterparts of DTI findings, *wt* or *TDP-43*^*G298S*^ mice were sacrificed at 6–8 months (*N* = 3 and *N* = 3, respectively) or at 14–17 months of age (*N* = 3 and *N* = 4, respectively) by perfusion fixation (performed as previously reported; [43]). The brain was then dissected out, post-fixed in 4% PFA in PBS for 24 h at 4 °C, cryoprotected in 30% Sucrose in PBS, embedded in OCT (TissueTek, Fischer Scientifics) and sectioned at 40 μm thickness in a Leica Cryotome (Leica Cryotom AG Protect, CM 1950). For each brain, 6 sections were considered for further processing, starting 0.6 frontal of bregma and every 40 μm thereafter for 500 μm; all sections were processed together to ensure the homogeneity of the staining procedure. Immunostaining was performed as previously reported [43]: briefly, brain sections were blocked and permeabilized in 3%BSA/2%FBS/0.1% Triton X-100 for 1 h at 24 °C and then incubated for 48 h a 4 °C with anti NeuN antibody (diluted 1:100 in blocking buffer); thereafter, the sections were washed in PBS (3 × 30 min, 24 °C), exposed to secondary antibody (donkey anti mouse conjugated with Alexa647 fluorochrome, 1:1000 in blocking buffer, 2 h at 24 °C), washed once again (3 × 30 min in PBS) and mounted with ProLong Gold (ThermoFisher). Each brain section was imaged in its entirety using a Leica microscope (Leica Live Cell Imaging Microscope DMI 6000B equipped with a 10X objective) with 12-bits image depth; imaging parameters were set to avoid over- or undersaturated areas and were kept constant when imaging the different sections.

For the quantification, each image was imported in ImageJ, subject to background subtraction (rolling-ball mode), thresholded at 130 (grey value in arbitrary units ranging between 0 and 4095) and contrast-enhanced (range 130–1300). A rectangular region of interest (approx. Size 2.5*10^5^ μm^2^) was manually located in correspondence of the layer V of primary motor cortex, identified based on the anatomical landmarks according to the Allen Reference Brain Atlas (available at https://mouse.brain-map.org/static/atlas). Within the region of interest, neurons were manually counted by an experimenter blind to the genotype. Neuronal counts were obtained for left and right motor cortex; sites containing processing or staining artifacts were excluded. For each mouse, average neuronal density was calculated from at least 4 artifact-free sections. Statistical analysis was performed using two-way ANOVA (age and genotype) with Tukey-corrected post-hoc comparison. Statistical significance was set at *p* <  0.05 after correction for multiple comparisons.

## Results

### Whole brain-based spatial statistics show loss of FA in motor cortex of TDP-43 mice

The experimental design included two sets of pre-specified analysis: first, the cross-sectional comparison of *wt* and *TDP-43*^*G298S*^ cohorts at baseline and at follow-up and second, the longitudinal comparison of *wt* and *TDP-43*^*G298S*^ mice compared to their baseline scans. Based on previously published data on longitudinal progression of the *TDP-43*^*G298S*^ mice, we set the timepoint for the baseline at approx. 8–9 months of age, when grip strength is still comparable in *TDP-43*^*G298S*^ and control mice and the timepoint for the follow-up at on average 14 months of age, when *TDP-43*^*G298S*^ mice display a significant decrease in grip strength [43]; within this timeframe the performance on the running wheel is significantly reduced in *TDP-43*^*G298S*^ mice compared to *wt* mice at both timepoints but does not decline between the timepoints and mice do not yet show weight loss or visible amyotrophy. Thus, the experimental design is set to identify abnormalities that may correlated with the onset of disturbances in the grip strength in *TDP-43*^*G298S*^ mice.

The FA maps for *wt* mice displayed remarkable stability over time. No significant clusters in the comparison of baseline and follow-up FA maps of *wt* mice were detected.

At baseline at 9 months of age *TDP-43*^*G298S*^ and *wt* did not display any significant regional differences across the whole brain volume. However, at the follow-up at 14 months of age the comparison of *TDP-43*^*G298S*^ vs. *wt* mice revealed a significant reduction in FA in a cluster of voxel spanning the boundary between the primary motor cortex (M1) and the adjacent secondary motor cortex (M2) in the right hemisphere (Table 1).

**Table 1:** Clusters of significant FA reduction from WBSS for comparisons *TDP-43*^*G298S*^ vs *wt* at follow-up, *TDP-43*^*G298S*^ at follow-up vs baseline and ΔFA differences for *TDP-43*^*G298S*^ vs *wt*. BREGMA coordinates are given in coronal/horizontal/sagittal orientation

No.	Size	BREGMA (cor./hor./sag.)	*p*	Anatomical localization
Follow-up: *TDP-43*^*G298S*^ vs wt				
1	1240	1.3 / -2.6 / 1.5	< 0.000001	M1 and M2
*TDP-43*^*G298S*^: follow-up vs baseline				
2	9696	−1.3 / -1.5 / 0.0	< 0.000001	retrosplenial granular cortex
3	4820	0.0 / -1.5 / 0.0	< 0.000001	cingulate cortex
4	4189	1.3 / -2.6 / 1.5	< 0.000001	M1 and M2
5	4128	−2.1 / -5.6 / -1.5	< 0.000001	posterior thalamic nucleus
Longitudinal: *TDP-43*^*G298S*^ vs wt				
6	942	1.2 / -1.5 / 1.5	< 0.000001	agranular insular cortex
7	613	1.2 / -4.6 / -2.5	< 0.000001	M1 and M2

Notably, when baseline and follow-up scans of the *TDP-43*^*G298S*^ mice were compared (so to obtain an intra-subject comparison and further decrease variability), a significant decline in FA was identified not only in the M1/M2 area but also in clusters located in the cingulate cortex, in the retrosplenial granular cortex, and in the posterior thalamic nucleus (Table 1).

**Fig. 2:**
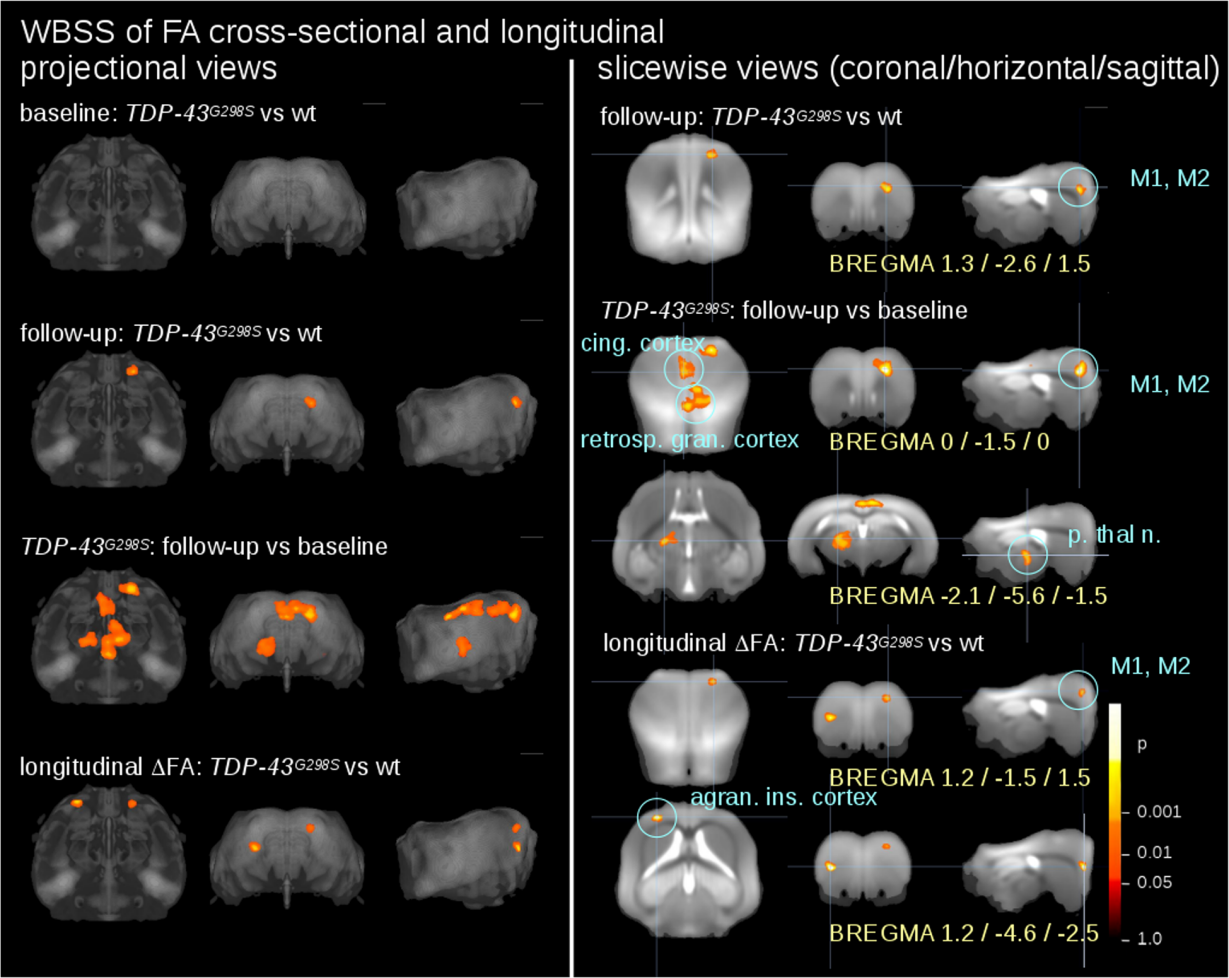
Whole brain-based spatial statistics (WBSS) for the comparison of FA values and for the comparison of ΔFA. Cross-sectional comparisons were performed for *TDP-43*^*G298S*^ mice vs *wt* at baseline and at follow-up and for *TDP-43*^*G298S*^ mice follow-up data vs baseline data; longitudinal comparison (ΔFA) was performed for *TDP-43*^*G298S*^ mice vs *wt*. Left panel: projectional views. Right panel: slicewise presentation of results clusters. Hot colors indicate an FA reduction or an increased ΔFA, respectively. M1, M2 – motor area 1,2, p. thal. n. – posterior thalamic nucleus, cing. Cortex – cingulate cortex, retrosp. Gran. Cortex – retrosplenial granular cortex, agran. Ins. cortex – agranular insular cortex

Longitudinal voxelwise differences of *TDP-43*^*G298S*^ mice were compared to longitudinal voxelwise differences of *wt* mice. ΔFA was linearly normalized to an identical time-interval prior to statistical comparison. The WBSS of ΔFA for *TDP-43*^*G298S*^ vs *wt* mice not only confirmed the loss of FA in the M1/M2 cluster, but also demonstrated an additional cluster in the agranular insular cortex (Fig. 2, Table 1).

### Region-of-interest analyses of the lateralization of the FA loss in motor cortex

**Fig. 3:**
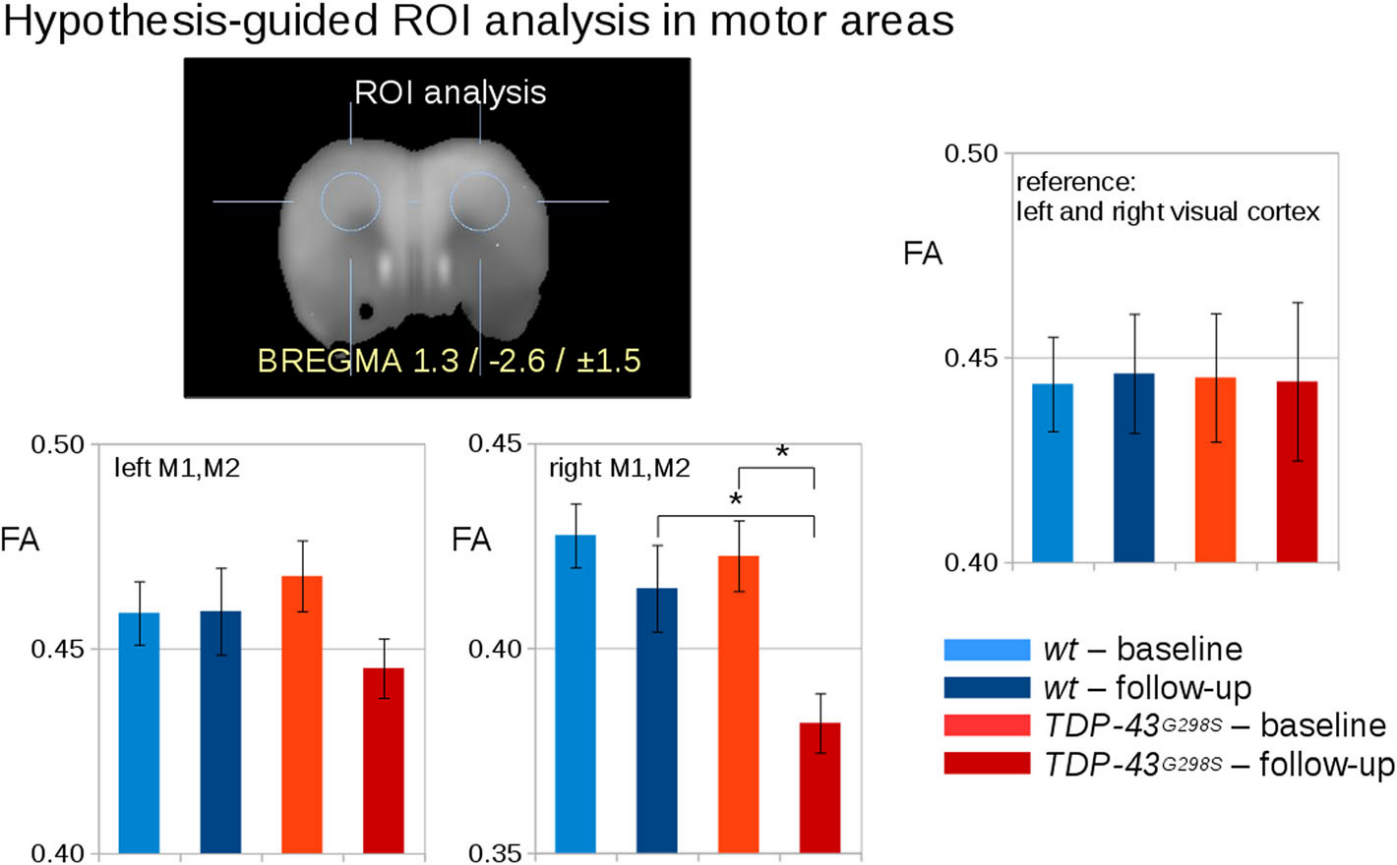
Hypothesis-guided analysis of FA values for *TDP-43*^*G298S*^ and *wt* mice at baseline and at follow-up. Left: Spherical ROI analysis in the motor areas M1,M2 (BREGMA -1.4 / 1.7 / ±1.5) show a bihemispherical reduction of FA values in motors areas M1 and M2 . Right: ROI based FA analysis in the visual cortex (BREGMA -4.4 / 1.7 / ±3.0). *significance *p* <  0.05. Error bars are given as standard error of the mean (SEM)

Since the WBSS data detected a hemispheric asymmetry in the decrease in FA in *TDP-43*^*G298S*^ mice, which may be a statistical artifact of the whole-brain comparison, a hypothesis-guided ROI analysis was performed with spherical ROIs located either in the right M1 and M2 or in the left M1 and M2 areas. Confirming the WBSS data, a significant FA reduction was observed at follow-up for the *TDP-43*^*G298S*^ mice in the right motor areas, whereas the left motor areas showed a trend (*p* = 0.1) for FA reduction (Fig. 3).

### Diffusion metrics reveals a selective increase in mean and radial diffusivity in motor cortex of TDP-43^G298S^ mice

Although FA changes could be reliably identified in the motor cortex of *TDP-43*^*G298S*^ mice, these findings alone do not provide information on the underlying the histopathological substrate. We then explored whether the FA loss could be due to changes in the microstructure of the motor cortex. To this aim, we considered the diffusivity metrics of the motor cortex in *wt* and *TDP-43*^*G298S*^ mice. While FA is sensitive to microstructural changes, it does not indicate a specific type of lesion; on the other hand, among the FA metrics, MD is an inverse measure of the membrane density, AD tends to be strongly affected by axonal injury whereas RD is sensitive to white matter damage due to demyelination and less to changes in the axonal density or size [19, 37, 41, 44].

**Fig. 4:**
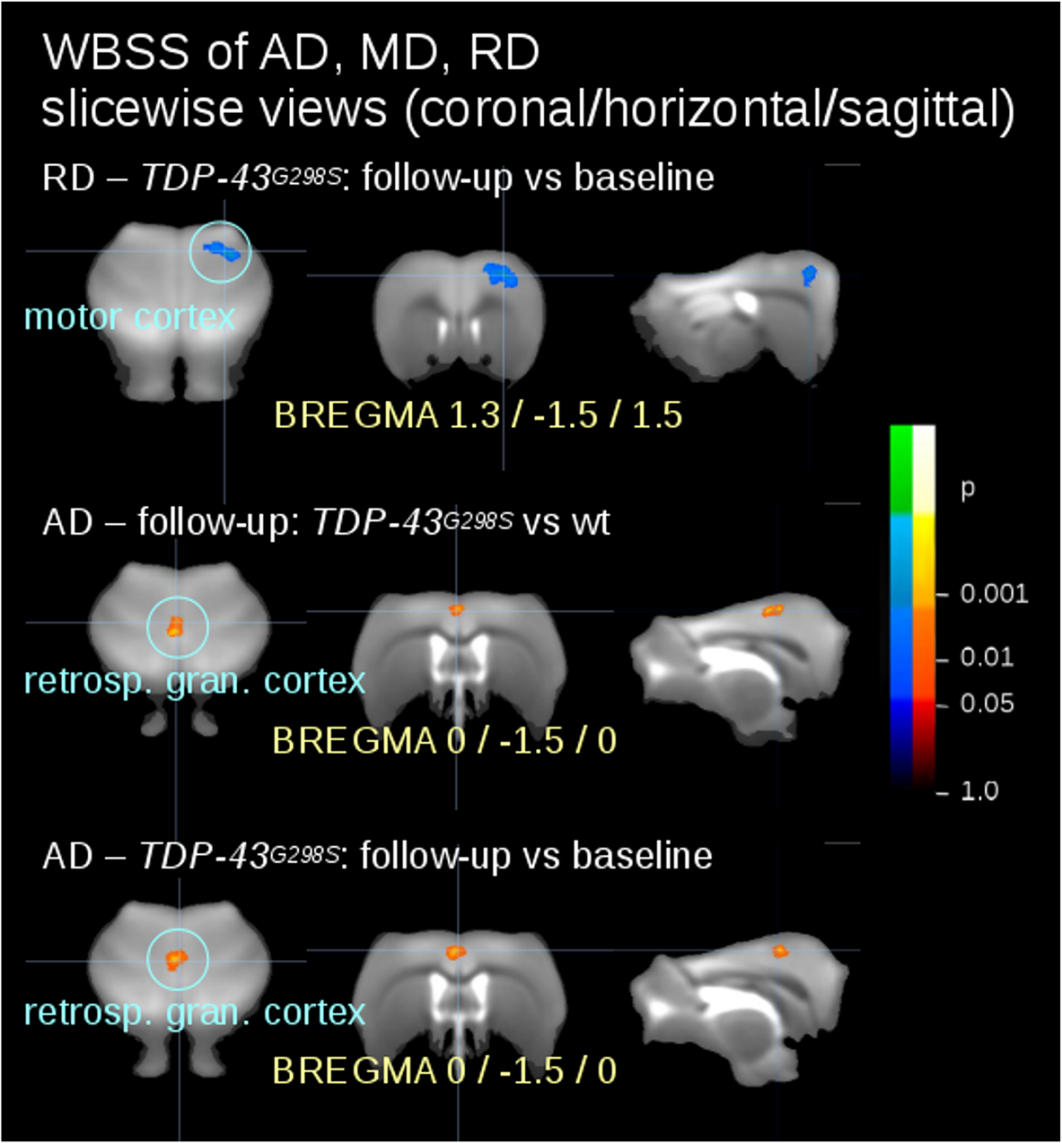
Whole brain-based spatial statistics (WBSS) for the comparison of AD, RD, MD values. Cross-sectional significant alterations were found for *TDP-43*^*G298S*^ mice vs *wt* at baseline and at follow-up and for *TDP-43*^*G298S*^ mice follow-up data vs baseline data. Slicewise presentation of results clusters. Hot colors indicate a reduction, cold colors an increase. Retrosp. gran. Cortex – retrosplenial granular cortex

Although no difference was detected in the comparison of *wt* and *TDP-43*^*G298S*^ mice at baseline (in agreement with the FA values), the whole-brain voxelwise comparison of AD, MD and RD in *wt* vs *TDP-43* mice at follow-up revealed a significant increase of RD in the motor cortex, whereas clusters on decreased AD were detected in correspondence of the retrosplenial cortex (Fig. 4).

Thus, these findings supported the hypothesis that FA alterations may be due to selective disturbances of the microstructure in the motor and extra-motor cortex of *TDP-43*^*G298S*^ mice.

### Tractwise fractional anisotropy reveals no damage to corticospinal and transcallosal fibers

**Fig. 5:**
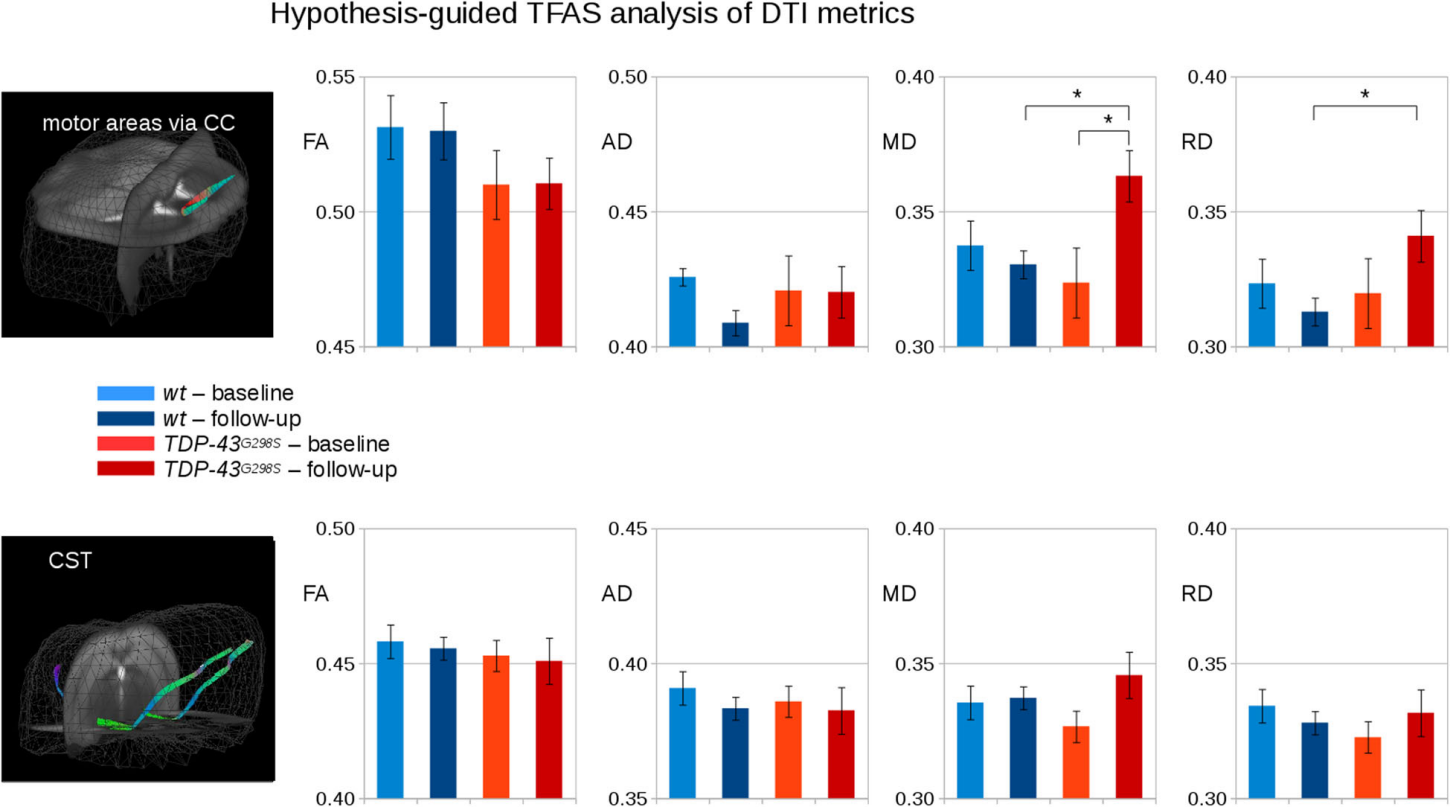
Hypothesis-guided TFAS analysis of DTI metrics (FA, AD, MD, RD) for *TDP-43*^*G298S*^ and *wt* mice at baseline and at follow-up. Upper panel: FT for the connection of motor areas via the corpus callosum (CC). Lower panel: FT for the corticospinal tract (CST). *significance p <  0.05. Error bars are given as standard error of the mean (SEM). AD, MD, RD are given in 10^-3^ mm^2^/s

Primary and secondary motor cortex give origin to multiple fiber tracts, connecting them either to sub-cortical and spinal targets (i.e., corticostriatal and corticospinal tracts – CST) or to other cortical areas (e.g., contralateral homonymous regions; [31]). Since degeneration of the CST is a hallmark of ALS in human subjects (and detectable by DTI; [16, 17]), we investigated whether the regional FA decrease observed in motor areas was specifically correlated with the disturbance of a subset of efferent fibers from motor areas. To this aim, TFAS was performed for the CST and in the transcallosal projections between ipsi- and contralateral motor cortices. Despite the detection of FA loss in the motor cortex, values of MD, RD and AD in the CST remained comparable in *wt* and *TDP-43*^*G298S*^ mice at all timepoints, indicating that the disturbances observed in the whole-brain analysis are not due to CST degeneration. On the other hand, a distinct change in MD and RD values was detected in trans-callosal fibers (Fig. 5). Thus, changes in cortical FA metrics are correlated with alterations in a subset of projection neurons not including those giving rise to the CST.

### Histological analysis of neuronal density reveals neuronal loss corresponding to FA loss in primary motor cortex

**Fig. 6:**
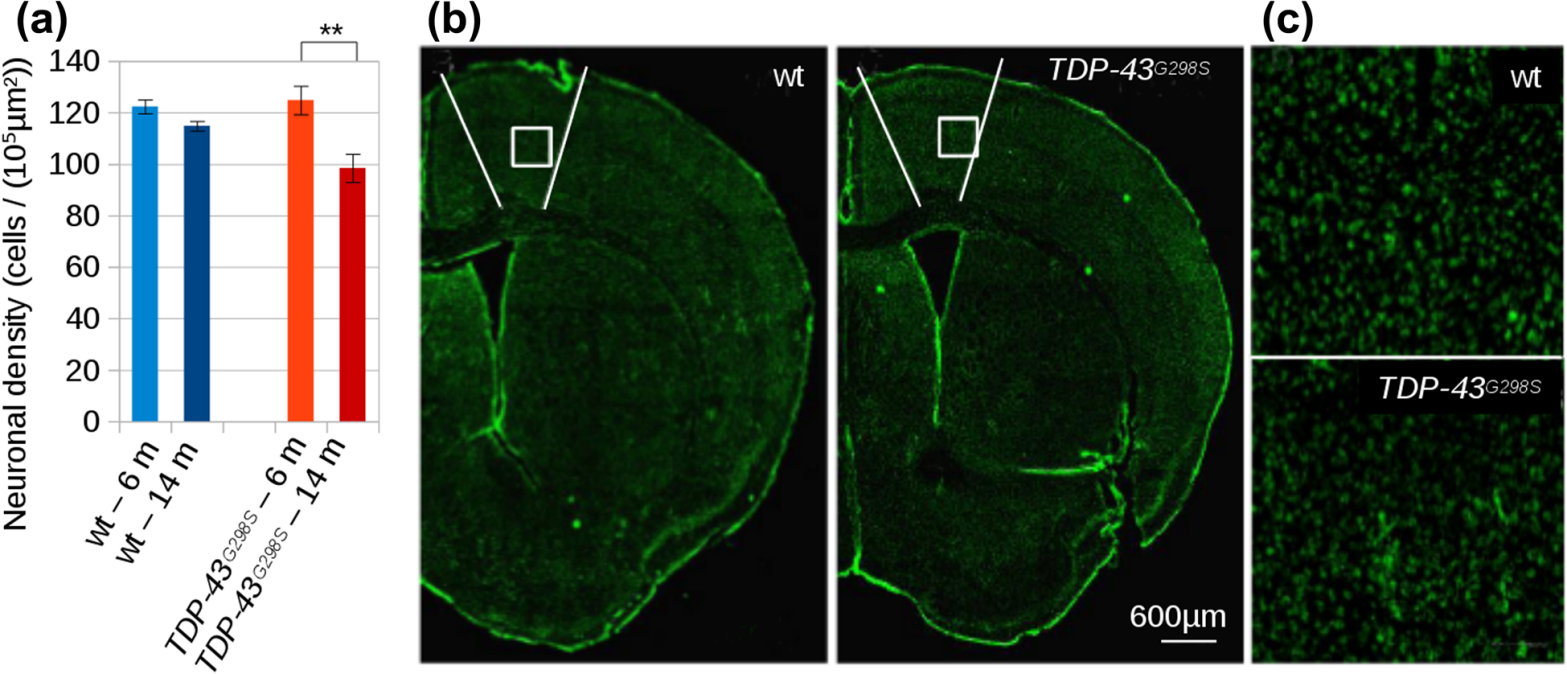
Neuronal counts in Layer V of M1 in *TDP-43*^*G298S*^ and *wt* mice at two timepoints. **a** The density of NeuN+ cells was measured in M1 of *wt* and *TDP-43*^*G298S*^ mice in distinct cohorts (at 6–8 months and 14–17 months of age). Whereas *wt* mice do not display a significant decline in the density of neurons in layer V, *TDP-43*^*G298S*^ mice show a significant loss over time. **b** Representative coronar overview images (scalebar 600 μm) with M1 boundaries highlighted for *wt* and *TDP-43*^*G298S*^. **c** High magnification insets (scalebar 100 μm, white square in the overview images). ** *p* = 0.001; m = months

The anatomical basis underlying the microstructural changes (detected by FA) was investigated by evaluating the extent of neuronal loss in M1 in two samples of mice at two timepoints (independent of those subject to MRI imaging); the earlier timepoint was at 6–8 months of age (*wt* (*N* = 3) and *TDP-43*^*G298S*^ (*N* = 3)) and the later timepoint was at 13–17 months of age (*wt* (*N* = 3) and *TDP-43*^*G298S*^ (*N* = 4)). The density of NeuN+ cells in layer V of M1 (where large projection neurons, such as corticospinal neurons, are located) was assessed. Two-way ANOVA identified a significant effect of time (F (1,9) = 46.90, *p* <  0.001) and genotype (F (1,9) = 7.90, *p* = 0.020) and a significant interaction between the factors (F (1,9) = 14.75, *p* = 0.004); post-hoc analysis (Tukey correction for multiple comparisons) revealed comparable neuronal density in *wt* at the earlier and later timepoint (122.3 ± 2.7 NeuN+/10^5^μm^2^ at 6 months and 114.9 ± 1.9 NeuN+/10^5^μm^2^ at 14 months, *p* > 0.05), whereas *TDP-43*^*G298S*^ mice were comparable to *wt* mice at 6 months (124.8 ± 5.5 NeuN+/10^5^μm^2^, p > 0.05) but displayed a significant decline over time at 14 months (98.5 ± 5.5 NeuN+/10^5^μm^2^, *p* = 0.001) (Fig. 6). However, due to the small sample size for histological analysis, a test for a lateralization effect was not possible.

## Discussion

In the present work we have demonstrated (i) the technical possibilities for long-term longitudinal DTI analyses in murine models of neurodegenerative diseases, specifically in an ALS model using ultrahigh field (11.7 T) with 30 gradient directions, (ii) the appearance over time of FA abnormalities in the motor cortex as well cingulate, insular and retrosplenial cortices of mice with mutant *TDP-43* overexpression in neurons (Thy1.2 promoter) mice, and (iii) the dissociation between pathologic values of diffusivity indexes in motor cortex and normal values in the CST.

### Technical considerations: reproducible longitudinal data in murine DTI imaging

Although previous studies have demonstrated the broad feasibility of repeated DTI in murine imaging [6, 22], they were limited in spatial resolution and by the number of gradient directions; thus, previous analysis used segmentation techniques that did not allow a full-brain comparison and were restricted to identify changes in spinal cord [22]. In this study, the feasibility of microstructural analysis in a whole-brain spatial statistics analysis and in vivo fiber tracking of the DTI-based analysis of the mouse brain at the cohort level was demonstrated. Technically, this study has shown that, by spatial rescaling of the DTI data generated in high-resolution ultrahigh field with 30 gradient directions, the multiparametric analysis cascade as it has been established in human DTI data could be transferred to an analysis cascade for murine DTI data (in a whole-brain spatial statistics analysis, not previously reported in murine ALS models). Moreover, due to scanning at the ultrahigh field of 11.7 T, murine DTI data could be recorded with a high signal to noise ratio (SNR). Furthermore, rodent scans could be repeated frequently, that way offering the opportunity to obtain high quality repeated scans.

### TDP-43^G298S^ mice show disturbed cortical microstructure with spared corticospinal tract

Several studies in *SOD1* transgenic ALS murine models have revealed DTI disturbances in the spinal cord, findings interpreted in terms of axonal loss affecting the cortico-spinal tract and other long white matter tracts stretching between the brain and the spinal cord [6, 22, 40]. However, despite the well-accepted observation of DTI abnormalities in ALS patients [5, 17], technical limitations have prevented so far the analysis of DTI patterns in the brain of murine models over time. Having overcome these limitations, our findings suggest that *TDP-43*^*G298S*^ mice display involvement of the motor cortex and other cortical regions and this is seemingly not associated with involvement of the corticospinal tract (whose degeneration is an hallmark of ALS – [17]) but is rather linked to the involvement of local projection neurons (such as those projecting through the corpus callosum) and to intracortical microstructural change (highlighted by AD changes in the retrosplenial cortex). Importantly, these alterations are not detectable at a stage in which *TDP-43*^*G298S*^ mice show normal grip strength but appear once grip strength is strongly affected [43], suggesting that they may provide an imaging correlate of neurodegenerative changes. In fact, a neuronal loss in M1 was shown where DTI demonstrates significant changes in FA, indicating that degeneration of neurons (and of dendritic and axonal structures associated) may be one (not necessarily the only one) of the processes generating the abnormal FA signal. Thus, FA may be used as a non-invasive readout of the neurodegenerative process in *TDP-43*^*G298S*^ mice.

The pattern of abnormalities in the *TDP-43*^*G298S*^ mice, as detected at this stage of the disease progression, shows closer similarities with FTD patients rather than with ALS patients: whereas in the latter the involvement of motor cortex and CST is prominent early in disease progression [5, 17], in the former a subset of patients with intact CST can be identified [18]. FTD patients also show DTI changes in frontal callosal fibers [8] and in the thalamus [15]. It is important to note that the dissociation between grip-strength abnormalities, FA changes and normal CST metrics may be limited to the stage of progression under study (about 450 days of age); whether CST abnormalities might appear at later stages in the *TDP-43*^*G298S*^ mice, with the progression of the involvement of neuronal subpopulations, remains object of active investigation.

Furthermore, the cortical pattern of involvement observed in the *TDP-43*^*G298S*^ mice, involving primary and secondary motor cortices as well as agranular insula and retrosplenial cortices shows similarities with the involvement of frontal cortex, insular and posterior cingualate areas in FTD and the related Semantic Dementia [35, 36]. In agreement with this interpretation, FTD-like behavioural abnormalities (such as hyperphagic behavior) have been reported in unrelated *TDP-43* transgenic model [42]. Our findings include, intriguingly, an asymmetry in the involvement of the primary and secondary motor cortices (with statistically significant changes appearing in the right but not in the left hemisphere) in the *TDP-43*^*G298S*^ mice; it is worth noting that asymmetric cortical involvement is a well-known feature of FTD and ALS [14, 35] and that mice display a significant degree of lateralization in gene expression patterns and neurochemical properties (e.g., [11]). Analysis of larger cohorts may reveal if the asymmetry observed in this study is due to statistical noise or reflects an increased vulnerability of the right hemisphere in the *TDP-43*^*G298S*^ mouse model.

FA metrics can be sensitive to multiple pathological changes and, although they may offer insights into the ongoing processes, they cannot be unequivocally attributed to a single histological alteration [44]. The histological analysis of mouse brains suggested that at least one process revealed by FA analysis is the loss of neurons (and related structures) in the motor cortex. In addition, and more speculatively, the differential effect on RD (but not on AD) observed in motor cortex, and the simultaneous involvement of RD and MD in transcallosal fibers, may point toward the disproportionate involvement of myelin and myelinated fibers. Indeed, involvement of white matter in the form of white matter hyperintensities has been shown in FTD patients using FLAIR and T2 MRI imaging [33, 38] and, in a case for which pathology is available, has been related to activation of microglia rather than vascular changes [46] and may result from primarily axonal damage. On the other hand, the selective AD changes in retrosplenial cortex appear to point toward a selective disruption of axonal microstructure; the simultaneous involvement of retrosplenial and M1/M2 may suggest that the reciprocal circuit linking these two areas [45] may be selectively involved.

### Limitations

The study was not without limitations. The number of mice was comparatively small, especially in contrast to previous human studies with high subject numbers longitudinally [17], but are similar to other small animal studies. Thus, the significance levels are limited in a cohort of mice with limited sample size. A further limitation was that follow-up scans could not be acquired in all animals since single wt mice and *TDP-43*^*G298S*^ mice died during the course of the study. Moreover, mutant TDP-43 protein is expressed under control of the Thy1.2 promoter in this model, thus lacking expression in non-neuronal cells, while in the human situation TDP-43 is ubiquitously expressed, including glial cells; thus, our study may have revealed the cell-autonomous effects of mutant *TDP-43* expression, and more severe phenotypes may occur in case of neuronal and non-neuronal expression of the transgene.

## Conclusion

This study demonstrated the technical feasibility of high-resolution, longitudinal DTI for preclinical cohort studies in murine models at 11.7 T. DTI-based analysis of the brain of *TDP-43*^*G298S*^ mice showed alterations of motor areas M1/M2 (showing a temporal correlation with neuronal loss) which increase over time as well as a broader involvement of retrosplenial cortex and agranular insula. Taken together, the DTI data suggest that, within the ALS-FTD spectrum, the *TDP-43*^*G298S*^ mice show similarities to FTD-ALS syndrome rather than pure motoneuron disease. In this context, degeneration of transcallosal and intracortical projections (with notable sparing of the CST) appears to be the most prominent pathological process ongoing in *TDP-43*^*G298S*^ mice.

## Data Availability

The datasets generated during and/or analysed during the current study are not publicly available as the acquired DTI data are property of the University of Ulm, but are available from the corresponding author on reasonable request.

## Abbreviations

AD: Axial diffusivity
ALS: Amyotrophic lateral sclerosis
CC: Corpus callosum
CST: Corticospinal tract
DM: DTI metric
DTI: Diffusion tensor imaging
FA: Fractional anisotropy
FDR: False-discovery-rate
FT: Fiber tracking
FTD: Frontotemporal dementia
MD: Mean diffusivity
RD: Radial diffusivity
ROI: Region-of-interest
SEM: Standard error of the mean
SNR: Signal to noise ratio
TFAS: Tractwise fractional anisotropy statistics
TIFT: Tensor Imaging and Fiber Tracking
WBSS: Whole brain-based spatial statistics
*wt*: Wild type
